# Radiographic Measurement of Gastric Remnant Volume After Laparoscopic Sleeve Gastrectomy: Assessment of Reproducibility and Correlation with Weight Loss

**DOI:** 10.1007/s11695-021-05812-0

**Published:** 2021-11-19

**Authors:** Małgorzata Deręgowska-Cylke, Piotr Palczewski, Marcin Błaż, Radosław Cylke, Paweł Ziemiański, Wojciech Szeszkowski, Wojciech Lisik, Marek Gołębiowski

**Affiliations:** 1 1st Department of Clinical Radiology, Medical University of Warsaw, Warsaw, Poland; 2 Department of General and Transplant Surgery, Medical University of Warsaw, Warsaw, Poland; 3 2nd Department of Clinical Radiology, Medical University of Warsaw, Warsaw, Poland

**Keywords:** Laparoscopic sleeve gastrectomy, Upper gastrointestinal series, Gastric remnant volume, Weight loss

## Abstract

**Background:**

As a restrictive procedure, laparoscopic sleeve gastrectomy (LSG) relies primarily on the reduction of gastric volume. It has been suggested that an immediate postoperative gastric remnant volume (GRV) may influence long-term results of LSG; however, there are no consensus in this matter. The aim of this study was to assess the reproducibility of different radiographic methods of GRV calculation and evaluate their correlation with the weight loss (WL) after surgery.

**Methods:**

This retrospective study evaluated 174 patients who underwent LSG in the period from 2014 to 2017. Using UGI, GRV was measured with 3 different mathematical methods by 2 radiologists. Intraobserver and interobserver calculations were made. Correlation between GRV and WL were estimated with calculations percentage of total weight loss (%TWL) and percentage of excess weight loss (%EWL) after 1, 3, 6, 12, 18, and 24 months postoperatively.

**Results:**

During analysis of intraobserver similarities, the results of ICC calculation showed that reproducibility was good to excellent for all GRV calculation methods. The intraobserver reproducibility for Reader I was highest for cylinder and truncated cone formula and for Reader II for ellipsoid formula. The interobserver reproducibility was highest for ellipsoid formula. Regarding correlation between GRV and WL, significant negative correlation has been shown on the 12th month after LSG in %TWL and %EWL for every method of GRV calculation, most important for ellipsoid formula (%TWL – r(X,Y) = -0.335, *p* < 0.001 and %EWL – r(X,Y) = -0.373, *p* < 0.001).

**Conclusion:**

Radiographic methods of GRV calculation are characterized by good reproducibility and correlate with the postoperative WL.

**Graphical Abstract:**

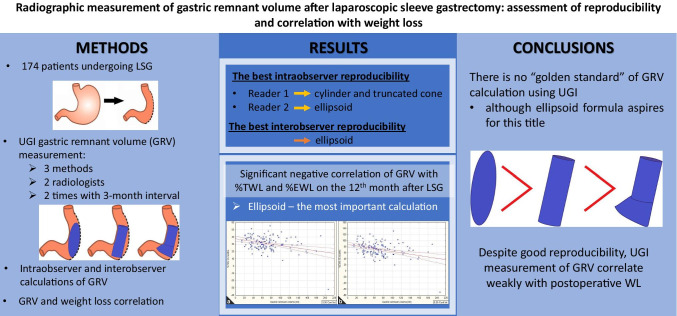

## Introduction

Bariatric surgery has proved safe and effective for treatment of patients with class II obesity (BMI 35–39.9) associated with obesity-specific comorbidities and with class III obesity (BMI ≥ 40) [1, 2]. In recent years, laparoscopic sleeve gastrectomy (LSG) and laparoscopic Roux-en-Y gastric bypass (LRYGB) have emerged as the most popular bariatric procedures [3]. LSG is perceived as one of the safest bariatric operations. A relatively simple surgical technique without need to create anastomoses, short learning curve, low rate of metabolic complications, and high success rate are its main advantages [4]. There are a lot of possible variables that may influence the success rate of LSG. Several factors associated with surgical technique have been studied, including volume of resected stomach, gastric remnant volume (GRV), and the size of a bougie used to calibrate gastric sleeve (GS) diameter [5–8]. GRV may be assessed either intraoperatively, by recording the amount of fluid used to expand the gastric remnant during methylene blue leak test, or with imaging studies: upper gastrointestinal study (UGI) or computed tomography (CT) [9–18].

UGI is a simple yet practical study that can be used to assess the gastric remnant anatomy, tightness, and volume. Based on literature review and experience gained in our department, we used three approaches to calculate GRV from UGI images based on the similarity of the GS to different geometrical figures [9, 11–13]. For years, also in our surgical center, UGI had been routinely performed after LSG to exclude a possible leak before oral diet was introduced. Recently, after introduction of new patient care protocols, such as Enhanced Recovery After Bariatric Surgery, and reports on relatively low diagnostic accuracy of UGI in leak detection, routine use of UGI has been questioned [19–22].

To the best of our knowledge, none of the previous publications compared different methods of calculating GRV from UGI images or assessed their reproducibility. Hence, it is difficult to pinpoint the optimal method of GRV calculation we decided to conduct a retrospective analysis of our database of UGI studies with a two-fold aim. The first objective was to identify the GRV calculation method offering the highest precision of measurements. The second objective was to analyze the relationship between GRV and weight loss (WL) and to assess whether the information on GRV obtained with UGI may justify its continued use in routine medical practice.

## Material and Methods

### Patients 

We conducted an analysis of radiological images and clinical data of patients qualified for a bariatric surgery program between the beginning of 2014 and the end of 2017. The inclusion criteria were based on the bariatric operation type (LSG) and the availability of an adequate radiological examination (UGI) performed on the 1st or 2nd days after surgery. In the analyzed period, 218 LSGs were performed. We had to exclude 44 patients from this study because of suboptimal quality of the radiological examination. Anthropometric characteristics of 174 patients included in our study are presented in Table 1.

**Table 1:** Anthropometric characteristics of the study group

	Mean	Min	Max	SD
Sex (% females)	74.71			
Age (years)	42.34	19.00	68.00	10.72
Weight (kg)	126.46	90.90	213.50	21.46
Height (cm)	169.30	153.00	192.00	8.46
BMI	44.01	34.55	69.81	6.00

### Surgical Technique

A thorough qualification for the bariatric procedure was performed in the outpatient surgical clinic. Indications for the surgery were BMI ≥ 40 or BMI 35–39.9 with a presence of obesity specific comorbidities [23]. Patients were encouraged to lose weight prior to surgery which has several advantages, mainly reduction of weight and volume of liver reduce risk of liver injury. Moreover, the lower the weight before the operation, the better the results of postoperative WL. Success in this endeavor did not exclude them from the operation, even if their BMI dropped below the level required for this surgery. Patients with a severe gastroesophageal reflux disease were disqualified from the surgery.

The surgery was performed laparoscopically utilizing 5 trocar ports. A laparoscopic liver retractor was deployed through the trocar below left costal margin. A 36 Fr bougie was used for calibration of the GS. The greater curvature of stomach was dissected with a laparoscopic energy device, and stomach resection was performed using a laparoscopic linear stapler. Resection began approximately 4 cm from the pylorus and was continued to the angle of His to completely resect stomach fundus. At the end of every operation, anastomotic leak test with methylene blue solution was conducted.

### Gastric Volume

**Fig. 1:**
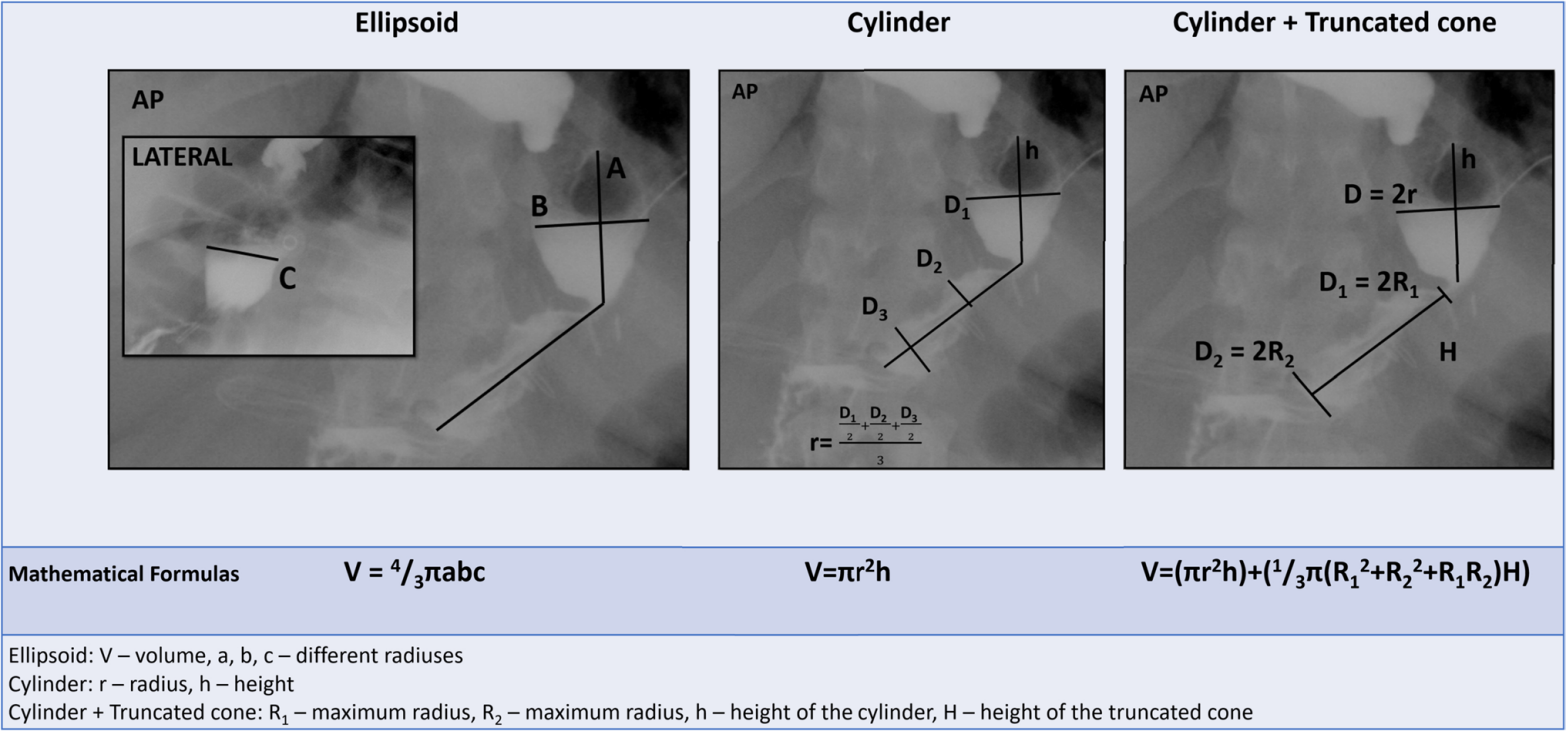
Different approaches to calculate gastric remnant volume

UGI was performed as a part of perioperative protocol on the 1st or 2nd days after surgery as an additional leak test before oral diet introduction. All UGI studies were performed on Ultimax Fluoroscopy System (Toshiba Medical Systems Corp., Tokyo, Japan). The distance between X-ray machine and X-ray table (on which patient lean on) was calibrated at 55 cm. When measuring the dimensions of gastric remnant, we assumed 40 pixels to be 10 mm. These settings were made in cooperation with Toshiba technician. These assessments were calibrated using radiopaque measuring object. In our study, every patient was evaluated on the same X-ray machine, using the same settings. The patient was given a cup of a water-soluble contrast medium (Ultravist 300, Bayer AG, Berlin, Germany), and while the patient was drinking it, the passage through the gastric remnant was examined under fluoroscopy. When the patient felt full (usually after drinking 50–70 ml of contrast medium), X-rays in anterior–posterior, oblique, and lateral projections were taken in an upright position. Images with the greatest distension of gastric remnant were chosen for GRV calculation. GRV measurements were performed twice by two independent radiologists in sessions separated by a 3-month interval. For each series of measurements, GRV was calculated using three methods based on the similarity of gastric remnant to geometrical figures: ellipsoid, cylinder, and a complex of cylinder (the upper part of the remnant) and truncated cone (the lower part). The mathematical formulas used for each model and technique of measurements are presented in Fig. 1.

### Weight Loss

Weight loss-related data collected were as follows: BMI, percentage of excess weight loss (%EWL), and percentage of total weight loss (%TWL) (Table 2). The calculation of ideal body weight was based on the optimal BMI equal to 25. Surgical success was defined as %EWL ≥ 50%. The data were collected on routine follow-up visits scheduled for the 1st, 3rd, 6th, 12th, 18th, and 24th months after surgery. The measurements were performed using TANITA SC 330 body composition analyzer (Tanita Corporation, Tokyo, Japan). The availability of patients for follow-up visits at each time point is presented in Table 3.

**Table 2:** Methodology of weight loss calculations

Parameter	Formula
%TWL	$\frac{\mathrm{initial weight }\left(\mathrm{kg}\right)-\mathrm{current weight }(\mathrm{kg})}{\mathrm{initial weight }(\mathrm{kg})}\times 100$
%EWL	$\frac{\mathrm{initial weight }\left(\mathrm{kg}\right)-\mathrm{current weight }(\mathrm{kg})}{\mathrm{initial weight }\left(\mathrm{kg}\right)-\mathrm{ideal body weight }(\mathrm{kg})}\times 100$

**Table 3:** Weight loss results after LSG. *N* number of patients available at each follow-up visit

Month	%TWL		%EWL		Surgical success (%EWL ≥ 50%)	*N* (%)
	Mean	SD	Mean	SD		
1	10.03	3.65	23.04	9.32	0.64	157 (90.2)
3	18.48	4.28	41.14	11.49	25.0	104 (59.7)
6	26.60	6.10	60.23	15.32	76.8	125 (71.8)
12	31.24	9.98	71.31	23.55	88.37	129 (74.1)
18	34.79	12.49	76.56	28.64	89.47	57 (32.7)
24	35.47	9.84	79.51	21.87	97.96	49 (28.1)

### Statistical Analysis

Continuous variables were expressed as means ± standard deviation (SD). Intraclass correlation coefficients (ICC) were used for the evaluation of intra- and interobserver measurement repeatability. ICC estimates and their 95% confidence intervals were calculated based on a mean-rating (*k* = 2), absolute-agreement, 2-way mixed-effects model. ICC intra- and interobserver agreement measures were interpreted according to guidelines given by Koo and Lee: poor, below 0.5; moderate, between 0.5 and 0.75; good, between 0.75 and 0.9; and excellent, above 0.9 [24]. The Pearson’s correlation coefficient (*r*) was used to explore the relationship between GRV and WL. For these estimations, mean value of every GRV measurement was calculated. The comparison of postoperative GRVs between patients with and without WL success was assessed using parametrical unpaired *t*-test. Statistical significance was set at *p* < 0.05.

For statistical analysis, we used Statistica version 13.3.0 (TIBCO Software Inc., Palo Alto, CA, USA; http://statistica.io.stica; 2017) and MedCalc Statistical Software version 19.6 (MedCalc Software Ltd., Ostend, Belgium; https://www.medcalc.org; 2021).

## Results

Mean values and SDs of GRV calculated at each session by both readers are presented in Table 4.

**Table 4:** Estimates of mean gastric remnant volumes performed by two researchers

	Calculation method	I Session (ml)	II Session (ml)
Reader I	Ellipsoid	66.10 ± 38.41	72.20 ± 43.83
	Cylinder	59.85 ± 33.18	60.15 ± 34.17
	Cylinder and truncated cone	66.34 ± 38.74	67.74 ± 40.12
Reader II	Ellipsoid	74.42 ± 39.25	72.72 ± 37.42
	Cylinder	63.59 ± 33.27	62.76 ± 31.72
	Cylinder and truncated cone	67.04 ± 36.30	65.42 ± 33.34

The results of ICC calculation reported in Table 5 show that reproducibility was good to excellent for all GRV calculation methods. The intraobserver reproducibility for Reader I was highest for cylinder and truncated cone formula (0.896) and for Reader II for ellipsoid formula (0.972). The interobserver reproducibility was highest for ellipsoid formula (0.822 and 0.765 for the first and second measurement, respectively).

**Table 5:** ICC results

	Intraobserver reproducibility		Interobserver reproducibility	
	Reader	ICC	Measurement	ICC
Ellipsoid	I	0.873	**I**	**0.822**
	**II**	**0.972**	**II**	**0.765**
Cylinder	I	0.892	I	0.721
	II	0.950	II	0.710
Cylinder and truncated cone	**I**	**0.896**	I	0.704
	II	0.921	II	0.725

The best results are bolded

**Fig. 2:**
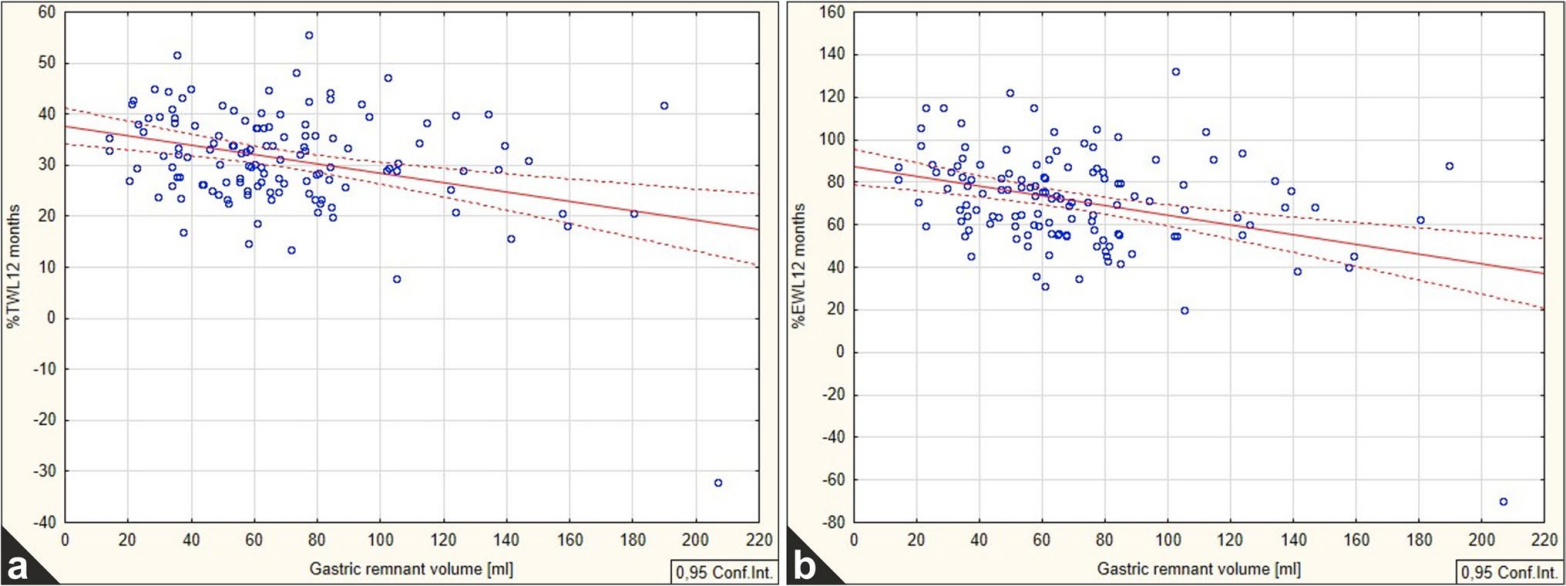
Correlation between gastric remnant volume (calculated using ellipsoid formula) to %TWL (**a**) and %EWL (**b**) on the 12 months of the follow-up

The dynamics of WL after LSG are presented in Table 3. Surgical success was achieved in 25% of patients at 3 months after surgery and in 76.8% and 88.4% at 6 and 12 months, respectively. Due to a significant dropout rate at the later follow-up visits (67% and 72% at 18 and 24 months, respectively), we decided not to include those data into statistical analysis. The correlations between GRV and WL are presented in Table 6. At the 1st and 2nd follow-up visits, a weak positive correlation was observed between GRV and both %TWL and %EWL. Following the 12-month follow-up, the correlation remained weak; however, it shifted to negative. The negative correlation between GRV and WL at 12 months was most pronounced for ellipsoid formula (%TWL – r(X,Y) = -0.335, *p* < 0.001 and %EWL – r(X,Y) = -0.373, *p* < 0.001) (Fig. 2). The remaining correlations were non-significant. The patients without successful WL at 12 months after surgery showed statistically higher postoperative GRVs (Table 7).

**Table 6:** Correlation between gastric remnant volume and weight loss

		%TWL		%EWL	
Formula	Month	r(X,Y)	*p*	r(X,Y)	*p*
Ellipsoid	1	**0.166**	**0.037**	0.109	0.175
	3	0.040	0.686	0.022	0.822
	6	-0.133	0.138	-0.162	0.071
	12	**-0.335**	** < 0.001**	**-0.373**	** < 0.001**
Cylinder	1	**0.166**	**0.037**	0.112	0.163
	3	0.082	0.406	0.063	0.523
	6	-0.149	0.097	-0.155	0.083
	12	**-0.202**	**0.021**	**-0.244**	**0.005**
Cylinder and truncated cone	1	**0.174**	**0.029**	0.116	0.147
	3	0.070	0.480	0.058	0.560
	6	-0.164	0.068	-0.175	0.051
	12	**-0.264**	**0.002**	**-0.302**	** < 0.001**

Statistically significant results are bolded

**Table 7:** Impact of GRV on the surgical success of the bariatric operation on the 12 months of follow-up

	Mean GRV ± SD (ml)		t	*p*
	Weight loss success group	No weight loss success group		
Ellipsoid	67 ± 39.339	88 ± 38.485	-3.950	** < 0.001**
Cylinder	57 ± 30.856	74 ± 33.755	-3.901	** < 0.001**
Cylinder and truncated cone	62 ± 34.281	81 ± 36.700	-4.069	** < 0.001**

Statistically significant results are bolded

## Discussion

The topic of correlation between GRV and WL after LSG has been covered in several articles. In radiological departments, GRV can be calculated at the basis of UGI or CT. Until recently, UGI after LSG was routinely used also to evaluate GS anatomy and gastric emptying and detect possible complications [25]. We decided to analyze GRV according to UGI results because this examination was previously a part of a standard protocol in all bariatric patients in our Surgery Department.

Our report shows that there is no one obvious way of calculating GRV after LSG. This is dependable on the examiner’s experience and preferences. Although formula for ellipsoid showed the highest repeatability between two investigators, it cannot be recommended as the method of choice in GRV calculation.

To our knowledge, this is the first study comparing reproducibility of different GRV calculation methods. In our Radiology Department, we used the formula for ellipsoid for the calculation of GRV so far. When comparing results of ellipsoid calculations, we found extremely good results in one of observers (Reader II), whereas for the other observer, results showed the smallest reproducibility among all the methods. Reader I showed the best results when calculating GRV as cylinder and truncated cone, which was the least favorable formula for the Reader II. Analysis of interobserver results showed that ellipsoid formula calculations demonstrate the highest reproducibility rate among all the others. This could be a consequence of it being the easiest method of all to calculate (it requires the lowest number of measurements). These results, although presenting high rate of repetitiveness, cannot lead to unshakeable conclusions and point on specific mathematical formula of GRV calculation as a method of choice for every institution. Each bariatric center should establish the most suitable approach of GRV calculation on their own, if they decide to perform such an analysis.

In this study, we analyzed a relation between GRV and WL. For the ellipsoid shape formula, we found statistically significant negative correlation for %TWL and %EWL at the 12th month after the operation. Interpretation of this result is that the lower the GRV calculated with ellipsoid formula, the higher the WL for the patient. This was the strongest result among the three different mathematical formulas for GRV calculations. Our estimations showed also a statistically insignificant positive correlation in %EWL and %TWL for 1st and 3rd and %TWL for 24th month. %TWL on the 1st month of follow-up showed statistically significant positive result. This could be due to the edema present in newly created GS that disturbs the calculations for the 1st and 3rd month results. Positive correlation of GRV with WL at 24th month of follow-up can be connected with a long period of observation time leading to decrease in compliance of patients after bariatric surgery. Pomerri et al. and Barbiero et al. used ellipsoid formula for the calculation of the gastric fundus volume only [9, 26]. They found no correlation between this parameter and postoperative WL. Pomerri et al. estimated GRV of 28 patients on the 2nd or 3rd day postoperatively [9]. The results showed no correlation between GRV and percentage of excess BMI loss (%EBMIL) after 3, 6, and 12 months after LSG. However, in 57 patients, sleeve voiding after the operation was assessed. WL in patients with fast sleeve voiding was significantly higher in comparison with slow sleeve voiding group. Barbiero and colleagues also tried to find a relationship between WL and gastric fundus volume measured using formula for ellipsoid [26]. Examined group consisted of 49 patients who underwent LSG, but their WL was unsatisfactory or they suffered from side effects of the operation like dysphagia, nausea, or vomiting. According to UGI, two groups were distinguished—13 patients with a proximal gastric pouch and 36 patients without gastric pouch. Statistical analysis showed no significant differences between these two groups that led to the conclusion that presence of proximal gastric pouch does not influence WL.

In our study, interpretation of results of GRV calculated as a cylinder presented statistically significant negative correlation for %TWL and %EWL at the 12th month after the operation. It was the weakest correlation among all of the presented results. Similarly to the ellipsoid formula calculations, it also showed a statistically insignificant positive correlation in %EWL and %TWL for the 1st and 3rd and %TWL for the 24th month, with the exception of %TWL on the 1st month of follow-up which showed statistically significant positive result. A Ferrer-Marquez et al. presented a prospective study with 112 patients after LSG in which also a cylinder formula has been used for measurement of GRV [11]. UGI performed on the 1st month and a year after the operation (which was also a time of a follow-up) showed a significant increase in GRV. However, they found none association between increase in GRV and WL at 1-year follow-up. They draw a conclusion that there is no correlation between this rise of GRV and %EBMIL.

Last mathematical formula used in this study was a combination of cylinder and truncated cone. Calculations of correlation between GRV and WL showed significant negative correlation for 12 months after the operation. A positive correlation was found for %TWL and %EWL in the 1st and 3rd months of the follow-up. It was statistically significant for %TWL in the 1st month after the operation. Vidal et al. used this method to estimate GRV in UGI as well [12]. Forty-five patients included in this study had GRV calculated at the 1st and 12th months, and %EWL was evaluated after 3, 6, 12, and 18 months following LSG. They identified statistically significant increase in GRV, although found no differences in proportions between two components of the stomach mentioned above. When analyzing %EWL, they found no correlation with results on the 3rd, 6th, and 12th month follow-up visits, but this correlation was statistically significant only at the 18th month. The %EWL at 18 months was inversely correlated with reservoir volume changes at 12 months after LSG (*p* = 0.006). They concluded that there is an association between increase of GRV and lower WL in the 18-month follow-up. Using the same formula for volume calculation, Panella et al. analyzed correlation of GRV and WL after LSG [13]. In their prospective study of 50 patients, measurements of GRV based on UGI in the 1st month and after 1 and 5 years postoperatively were made. They found upward trend in GRV between time intervals. Conclusion of their analysis was that there was negative correlation between GRV and WL in the 1st year after LSG that was not observed at the 5th year of the observation.

Surgical success of bariatric operation is commonly described as loss of 50% or more of excessive weight. We used this value as a threshold in calculations of its correlation with GRV. In our study, we found that the mean GRV of patients who achieved surgical success in WL is significantly lower when compared to those who failed in this endeavor. This can lead to the conclusion that the lower the GRV, the greater effect of the bariatric procedure, but because of a high SD of these values, definitive statements should not be established.

Our study has several limitations. First of all, the measurement of GRV was made at one timepoint only. The increase in GRV over time may influence the results of LSG. Data on this problem is controversial. Vidal et al. found that increase in GRV 1 year after the operation results in worse WL effect no earlier than at the 18-month follow-up after the surgery [12]. On the other hand, Panella et al. observed similar correlation at 1-year follow-up, which disappeared at 5th year [13]. To the contrary, Braghetto et al. found no correlation of WL with the increase of GRV estimated using CT study [14]. Second limitation to our study is lack of correlation with CT, which is more accurate examination than UGI. Routine use of CT for GRV calculation is not clinically sound until low dose protocols with comparable doses to UGI are available. Follow-up visits are not obligatory in our clinic, so the high dropout rate on subsequent visits is another limiting factor in this study.

Analysis of intra- and interobserver calculations led to the conclusion that there is not a one method that could be applied and recommended in every bariatric center and could be called “golden standard” for GRV evaluation. Calculations presented in this study showed high reproducibility, but none of these formulas stood out as a definitively superior one. Although the fact that an ellipsoid formula showed slightly better reproducibility rate can point to this method as a preferable one, more studies have to be conducted before any final recommendations could be made. In our study, correlation of GRV with WL showed a significant negative correlation a year after the surgery. This bears a predictive factor, meaning that the lower GRV is obtained, the better WL results could be achieved. This correlation shows a significant negative value for 12 months after LSG. It is non-significant, negative for the 6th and 18th months, and even could be positive for the 1st, 3rd, and 24th months after the operation. On the other hand, statistically significant correlation between the GRV and surgical success of bariatric operation has been found, but these results were weakened by high values of SD. This shows that WL after LSG is a multifactorial phenomenon and it is difficult to predict using a single variable. Considering that UGI is not indifferent, pose some radiation hazard, and does not provide significant clinical correlations in longer observation, performing this examination routinely bears questionable advantages.
